# Multicentric Castleman's Disease in a Child Revealed by Chronic Diarrhea

**DOI:** 10.1155/2015/689206

**Published:** 2015-02-09

**Authors:** Sarra Benmiloud, Sana Chaouki, Samir Atmani, Moustapha Hida

**Affiliations:** Unit of Pediatric Hematology-Oncology, Department of Pediatrics, University Hospital Hassan II, Faculty of Medicine and Pharmacy, University Sidi Mohamed Ben Abdellah of Fez, Morocco

## Abstract

Multicentric Castleman's disease is a rare benign and unexplained lymphoproliferative disorder that is extremely uncommon in children. It presents with fever, systemic symptoms, generalized lymphadenopathy, and laboratory markers of inflammation. Its treatment is not standardized and its prognosis is poor. We report a novel case of multicentric Castleman's disease in a 13-year-old girl who had presented with chronic diarrhea as the only initial presenting symptom. The diagnosis of celiac or inflammatory bowel diseases was suspected, but two and a half years later, the diagnosis of multicentric Castleman's disease was brought following the appearance of abdominal mass whose biopsy revealed Castleman's disease in the plasma cell form. The outcome was favorable after treatment by corticosteroid, chemotherapy, and surgery. The occurrence of diarrhea as the initial symptom of multicentric Castleman's disease without lymph node involvement is very rare. This case report underlines the diagnostic difficulties and the long interval between onset and diagnosis when diarrhea occurs first.

## 1. Introduction

Multicentric Castleman's disease (MCD) is a poorly understood lymphoproliferative disorder that is rarely reported in children [1, 2]. It is characterised by peripheral lymphadenopathy with systemic symptoms and laboratory markers of inflammation. Its real incidence is unknown, and its prevalence is estimated to be less than 1/100,000. Most reported cases have been described in adult patients, with a peak of incidence in the third and fourth decade of life for localized forms and in the fourth and fifth decade of life for multicentric forms [1]. In children, Castleman's disease (CD) is predominantly diagnosed during the teenage years and it is usually a localized type with a slight predilection for girls [2]. The MCD is very rarely reported in children and represents only about 13% of Castleman's cases [2].

The point of special interest in our case is that clinical presentation is atypical and characterized by the occurrence of chronic diarrhea as the only initial presenting symptom. This case underlines also the diagnostic difficulties.

## 2. Case Report

A 13-year-old girl with no past medical history, complained for 2 years of chronic diarrhea with no other clinical signs, and this has necessitated several visits to pediatricians without improvement under symptomatic treatment. Due to the persistence of diarrhea associated with growth retardation (weight and height were at minus 2 standard deviations (SD)) with a bone age corresponding to 10 years; celiac disease was suspected; antitransglutaminase and antiendomysium antibodies were negative; jejunal biopsy with histological study was read as villous atrophy II. The patient was put on gluten-free diet for 6 months without improvement. The evolution was marked by the persistence of diarrhea and the appearance of an intermittent fever, ranging between 37.8 and 38.5°C, asthenia, anorexia, weight loss, night sweats, and a deterioration of the general state. Second gastric and duodenal biopsies with histological study were read to show a nonspecific chronic interstitial duodenitis. The search of Koch's bacillus in sputum and tuberculin skin test was negative. Six months later, the patient developed insidiously growing abdominal mass; lymphoma was suspected, so she was transferred to our department for evaluation.

**Figure 1:**
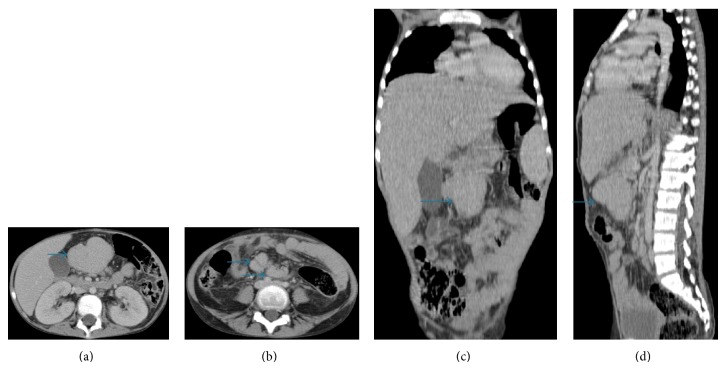
Thoracoabdominal-pelvic computed tomography injected in axial sections (a and b) with coronal (c) and sagittal (d) reconstructions demonstrating multiple adenomegalies and a mesenteric soft tissue mass.

Clinical examination at admission revealed a febrile patient at 38.5°C, in poor physical condition, pale, and with a growth retardation; her body weight was 24 Kg (minus 3 SD); and the body height was 138 cm (minus 3 SD). Abdominal palpation revealed a hepatomegaly (4 cm below the costal margin), a splenomegaly (2 fingerbreadth from the flange left costal), and a painless paraumbilical mass which was slightly mobile, measuring about 6 cm of diameter. The rest of the physical examination demonstrated lenticular inguinal and cervical lymph nodes. Abdominal ultrasound followed by thoracoabdominal-pelvic computed tomography revealed a hepatosplenomegaly, a multiple mesenteric lymph nodes (the largest measures 19 mm in diameter), and a soft tissue mass roughly rounded shape, well limited, enhanced moderately and homogeneously by the product of contrast and measuring 40 × 65 × 56 mm in diameter. This mass has an intimate contact with the gallbladder without signs of invasion, and a contact with the antropyloric region of the stomach, with a significant infiltration of the surrounding fat (Figure 1). The chest X-ray was normal. The biological investigations revealed microcytic hypochromic anemia (hemoglobin = 7 g/dL) (normal range 10.5–13.5 g/dL), thrombocytosis (platelet count = 489,000/mm^3^) (normal range 150000–450000/mm^3^), and elevated inflammatory markers: erythrocytes sedimentation rate (ESR) = 145 mm in the first hour (normal range < 16 mm), c-reactive protein = 252 mg/L (normal range 0–6 mg/L), fibrinogen = 7.1 g/L (normal range 2–4 g/L), ferritin = 527.9 *µ*g/L (normal range 20–250 *µ*g/L), serum iron = 0.10 mg/L (normal range 0.6–1.9 mg/L), hypoalbuminemia (22 g/L) (normal range 33–50 g/L), an increase of alpha 1 globulin (5.6 g/L) (normal range 1.2–4 g/L), alpha 2 globulin (14.4 g/L) (normal range 4–8 g/L), beta 2 globulin (6.7 g/L) (normal range 1–4 g/L), and hypergammaglobulinemia (37.5 g/L) (normal range 6–12 g/L). The insulin-like growth factor (IGF1) was low (26.5 ng/mL) (normal range 220–972 ng/mL) without abnormal secretion of growth hormone in the insulin stimulation test. The hepatic transaminases and lactate dehydrogenase were normal. The bacteriological, viral (Epstein Barr virus, cytomegalovirus, human immunodeficiency virus, hepatitis B and hepatitis C, and human herpes virus 8 (HHV8)), and parasite evaluation were negative. Immune and thyroid function tests were normal.

A scan-guided biopsy of the abdominal mass was performed. Microscopic examination revealed normal lymph node tissue architecture, with hyperplastic lymphoid follicles made of elements of variable size; in some areas they look like an onion bulb. In the interfollicular areas, a marked proliferation of plasma cells was identified. An immune-staining by the anti-CD20 and anti-CD3 shows a normal distribution of the lymphoid population. The anti-CD138 antibody shows a wealth of interfollicular tissue into mature plasma cells. These findings were compatible with the plasma cell form of CD. Because of the rarity of this disease, a study of the biopsy by another pathologist confirmed this result. The myelogram performed during general anesthesia was normal.

The patient received oral corticosteroid (prednisone) for a one month with a minimal response (20% the reduction of the size of the mass); thus she received chemotherapy based on 2 courses of VAMP (vinblastine, doxorubicin, methotrexate, and prednisone) that allowed a 62% reduction in the size of the mass, permitted to perform a total tumorectomy. The outcome was favorable with the disappearance of fever, night sweats, diarrhea, hepatosplenomegaly, and normalization of inflammatory markers. Currently we are at 26-month follow-up; the child is asymptomatic, starting to catch up the height and weight growth (minus 1.5 SD).

## 3. Discussion

This case illustrates the diagnostic difficulties when diarrhea occurs as the initial presenting symptom in MCD. This affection is characterised by peripheral lymphadenopathy (84%) with a mean of four sites involved and manifestations of multisystem involvement: fever, anorexia, weight loss, asthenia, weakness, night sweats, hepatosplenomegaly, skin rash, lung disorder, and kidney dysfunction. Sometimes, gastrointestinal symptoms may be encountered, such as diarrhea, vomiting or nausea, and less common, polyneuropathy, oedema, pleural or pericardial effusion, ascites, and so forth, [3, 4]. Typically there are also laboratory abnormalities: hypergammaglobulinemia, elevated inflammatory parameters, anemia, thrombocytosis, leucopenia, low serum albumin level, and, sometimes, elevated interleukin-6 (IL-6) [3]. MCD can be confused with malignant lymphoma; a definitive diagnosis requires an excisional biopsy. Histologically, MCD corresponds to the plasma cell type and is characterised by hyperplastic follicles, with marked proliferation of plasma cells in the interfollicular stroma, which is less vascular than in the hyaline-vascular variant.

The clinical course of MCD is variable; some patients may be largely asymptomatic or have spontaneous abatement of symptoms, but usually it may progress over several months or be episodic with recurrent exacerbations over a number of years [5]. The delay in diagnosis is often long because of clinical polymorphism and ignorance of the disease by pediatricians. In the case of our patient, it took two years and a half to make the diagnosis because we had diarrhea as the initial symptom without lymph node involvement. Initially, celiac or inflammatory bowel diseases were suspected with an impact on the growth of the child. But after the occurrence of an abdominal mass, lymphoma or CD was suspected. Reported gastrointestinal manifestations on CD in the literature are uncommon. The digestive tract may be secondarily involved and, in some cases, is responsible for the first manifestations of the disease. The gastrointestinal involvement was attributed to reactive amyloidosis or an intestinal lymphangiectasia [6, 7]. Rarely, there is colitis probably attributable to the immune dysregulation and cytokine induced intestinal epithelial cell apoptosis [8].

The aetiology of CD is unknown. Even if no underlying cause has been reported in children, it is generally believed that CD is an autoinflammatory disease resulting in an increase of IL-6 secretion. Several immunologic mechanisms have been proposed, including overproduction of IL-6 and HHV-8 infection [9, 10]. It is commonly thought to represent a defect in immune-regulation, resulting in an excessive proliferation of B lymphocytes and plasma cells in lymphoid organs. The opportunistic presence of HHV-8, favored by immune perturbations, and the direct pathogenic role of HHV-8, in association with dysregulation of cytokines, were suggested by demonstrating that HHV-8 is able to produce an IL-6 homologue, above all [10, 11]. This dysregulated overproduction of IL-6 by the affected lymph nodes is thought to be responsible for the systemic manifestations of the disease.

Treatment of MCD is not standardized. The optimal therapeutic approach is unknown. Diverse treatments are used, often in combination. MCD is too widespread to remove all affected nodes with surgery. Several types of treatment have been successful in some patients. Corticosteroid was used with amelioration of symptoms, but the effect is generally temporary with recurrence of symptoms likely on tapering or discontinuation of treatment [12]. In some cases, steroids alone can be sufficient for remission even though the treatment must often be prolonged [3]. In many cases, chemotherapy was used successfully in the symptomatic patients [3, 4]. Regimens against Hodgkin lymphoma are the most commonly used. These regimens use CVAD (cyclophosphamide, vincristine, doxorubicin, and dexamethasone) or CHOP (cyclophosphamide, doxorubicin, vincristine, and prednisolone). Radiation is sometimes used, but its role is uncertain except in localized form [13]. Adults with MCD have been successfully treated with agents including anti-IL-6 receptor antibody (tocilizumab), interferon-*α*, rituximab, and antivirals [1, 3, 4, 12]. Other treatments such as intravenous immunoglobulin, plasmapheresis, targeted agents (thalidomide and bortezomib), and autologous hematopoietic stem-cell transplantation have been used sporadically [4, 12]. In our case, we initially administered corticosteroids, but due to insufficient response, we gave the patient chemotherapy which allowed a good improvement with a regression of the size of the mass that was removed by surgery.

In MCD, despite treatment, the prognosis remains uncertain. The majority of children survive with persistence of disease. Death is either caused by infections or by the development of malignancy. About 20% of people with MCD develop lymphoma that usually grows fast and is hard to treat [3, 4, 12].

## 4. Conclusion

Pediatric MCD is a very rare benign lymphoproliferative disorder characterised by angiofollicular lymph-node hyperplasia. This affection is characterised by peripheral lymphadenopathy with manifestations of multisystem involvement and laboratory markers of inflammation. However, the diagnosis can be difficult because of the lack of clinical and radiological specificity. It is suggested by clinical presentation and confirmed by histology. The occurrence of chronic diarrhea as the only initial presenting symptom is atypical and can cause a delay diagnosis. MCD should be borne in mind in the differential diagnosis of patients with chronic diarrhea.
